# Supplementary figures and images for: The Making of Transgenic Drosophila guttifera
Source: Methods Protoc. 2020 Apr 27;3(2):31. doi: 10.3390/mps3020031 (PMC7359701; doi:10.3390/mps3020031)

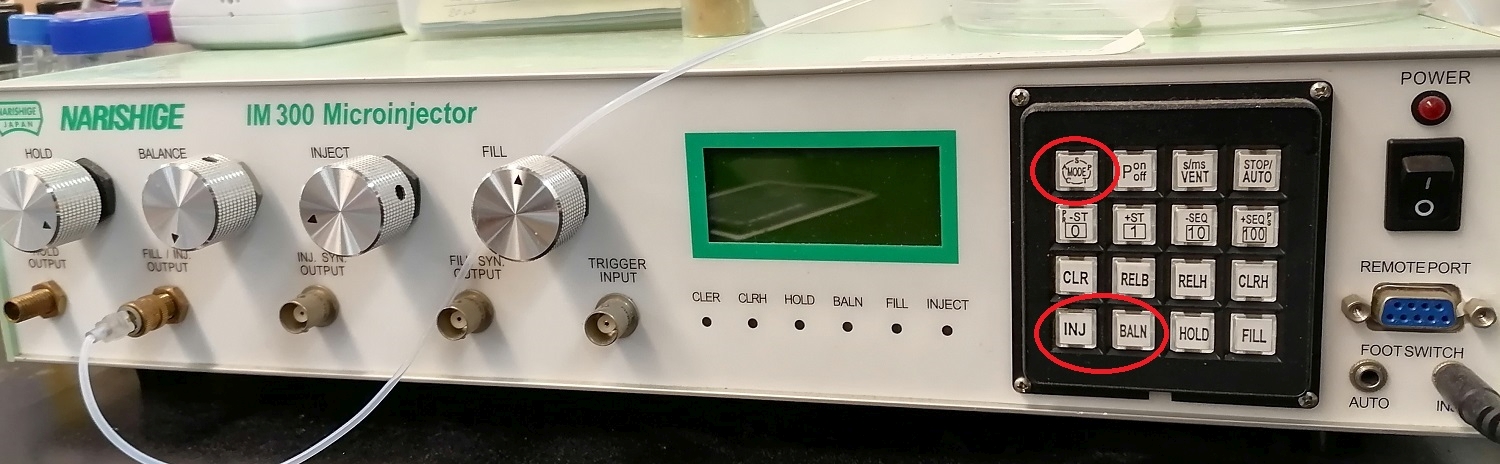

Supplement: Supplementary file 1 [file mps-03-00031-s001.zip › mps-781947-supplementary.jpg]
